# Local recurrence after rectal endoscopic submucosal dissection: a case of tumor cell implantation

**DOI:** 10.1007/s12328-013-0445-3

**Published:** 2013-12-21

**Authors:** Takashi Inoue, Hisao Fujii, Fumikazu Koyama, Tadashi Nakagawa, Kazuaki Uchimoto, Shinji Nakamura, Takeshi Ueda, Naoto Nishigori, Keijiro Kawasaki, Shinsaku Obara, Takayuki Nakamoto, Yoshiyuki Nakajima

**Affiliations:** 1 Department of Surgery, Nara Medical University, 840 Shijocho, Kashihara, Nara 634-8522 Japan; 2 Department of Endoscopy and Ultrasound, Nara Medical University Hospital, Kashihara, Japan

**Keywords:** Colorectal endoscopic submucosal dissection, Local recurrence, Tumor cell implantation, Intraluminal lavage

## Abstract

We report a case of local recurrence of cancer after rectal endoscopic submucosal dissection (ESD). A 52-year-old male underwent a curative resection with ESD for rectal intramucosal cancer. Seventy-four months after ESD, surveillance colonoscopy showed an elevated lesion on the ESD scar, suspicious of a recurrence. The patient subsequently underwent a low anterior resection (intersphincteric) with lymph node dissection. Pathology revealed a well-differentiated adenocarcinoma, similar to the ESD specimen. We suspected that the local recurrence was caused by implantation of tumor cells during the ESD, due to surgical manipulation performed with the tumor in an exposed setting for a long period of time.

## Introduction

Local recurrence after endoscopic resection of colorectal tumors is thought to be due to residual tumor left behind from the initial endoscopic resection [1]. To our knowledge, there have been no reported cases of local recurrence thought to be from implantation of tumor cells after endoscopic resection of colorectal tumors. We describe a case of local recurrence thought to be from implantation of tumor cells after endoscopic submucosal dissection (ESD).

## Case report

**Fig. 1:**
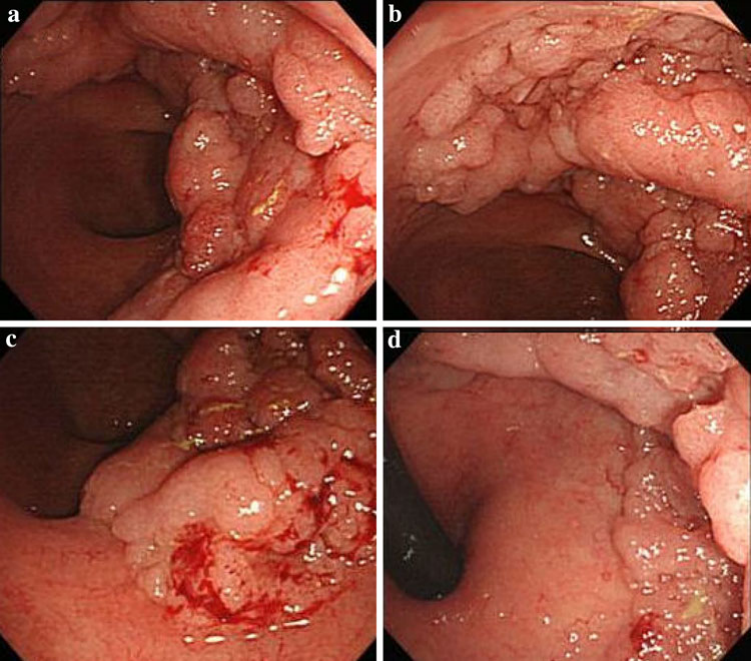
Colonoscopy showed a 0–IIa lesion, a granular-type laterally spreading tumor in the lower rectum

**Fig. 2:**
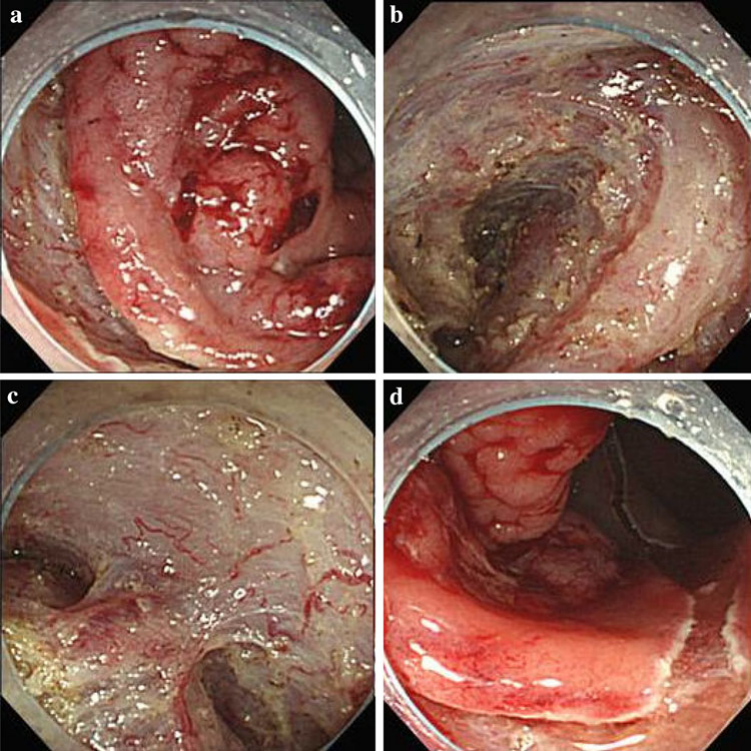
ESD procedure for a granular-type laterally spreading tumor in the lower rectum. **a** An incision was made into the mucosa distal to the tumor. **b** The submucosa was dissected just above the muscular layer toward the proximal side of the tumor. **c** Submucosal dissection was performed as the hood compressed the tumor. **d** The mucosal incision was extended proximally from the left and right sides to make a circumferential mucosal incision

**Fig. 3:**
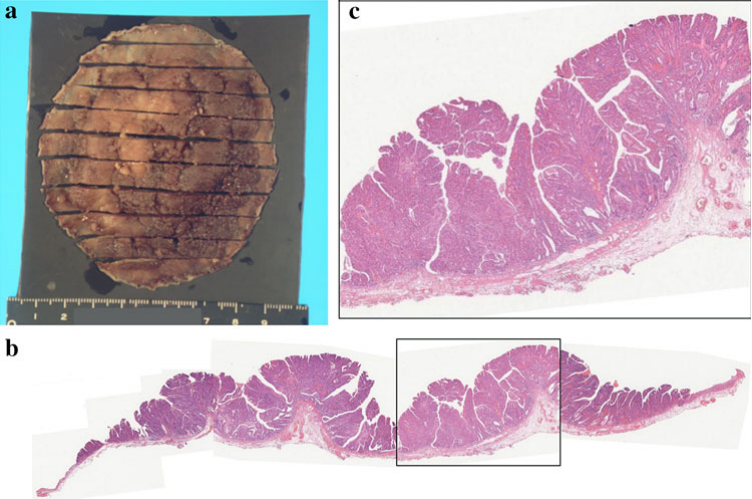
**a** Grossly, the tumor measured 60 × 65 mm and the lateral margin was negative. **b** Microscopic examination showed a well-differentiated adenocarcinoma, a negative vertical margin, a negative lateral margin, intramucosal cancer with no vascular invasion, and negative budding

**Fig. 4:**
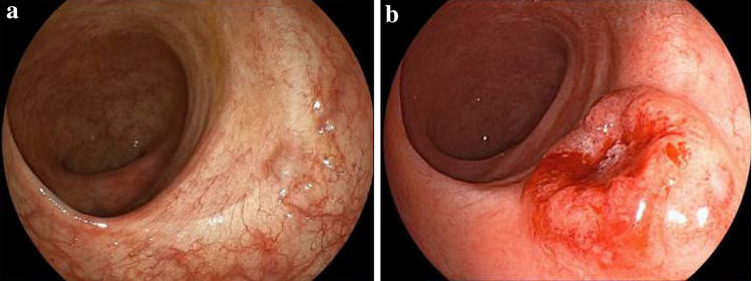
**a** Surveillance colonoscopy after 53 months showed no recurrence on the ESD scar. **b** Surveillance colonoscopy after 74 months showed an elevated lesion resembling a submucosal tumor on the ESD scar

**Fig. 5:**
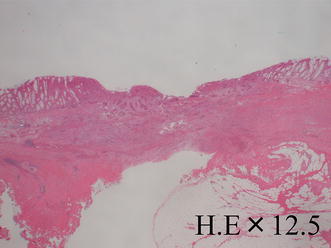
Microscopic examination revealed a well-differentiated adenocarcinoma, similar to the ESD specimen. The cancer was exposed to the mucosa at the central depression and infiltrated the muscular layer

The patient was a 52-year-old male. He had a positive screening fecal occult blood test, and a subsequent colonoscopy at an outside facility revealed a tumor in the lower rectum. He was admitted to our department for further treatment. Colonoscopy at our department showed a 0–IIa lesion, a granular type with lateral spread in the lower rectum (Fig. 1a–d). The procedure for ESD is as follows. A diluted hyaluronic acid solution was injected into the submucosa distal to the tumor. Subsequently, an incision was made into the mucosa distal to the tumor (Fig. 2a). Local injections were repeated as needed while the submucosa was dissected just above the muscular layer toward the proximal side of the tumor (Fig. 2b). Submucosal dissection was performed as the hood compressed the tumor (Fig. 2c). After adequate submucosal dissection was completed, the mucosal incision was extended proximally from the left and right sides to make a circumferential mucosal incision (Fig. 2d). Finally, the remaining submucosal layer was dissected and the tumor was resected en bloc (Fig. 3a). The procedure time required for ESD was 227 min. Grossly, the tumor measured 60×65 mm and the lateral margin was negative (Fig. 3a). The fixed specimen was cut into 33 slices at 2–3 mm intervals (Fig. 3a). Microscopic examination revealed a well-differentiated adenocarcinoma, a negative vertical margin, a negative lateral margin, intramucosal cancer with no vascular invasion, and negative budding (Fig. 3b, c). There was no evidence of histological damage to the muscularis mucosa by any cautery effect or air (Fig. 3b). Curative resection was therefore achieved, defined as satisfying all the following criteria based on the Japanese Society for Cancer of the Colon and Rectum Guidelines 2010 for the Treatment of Colorectal Cancer [2]—negative vertical and lateral margin, depth of submucosal invasion of <1,000 μm, negative vascular invasion, and negative budding. Fifty-three months after ESD, surveillance colonoscopy showed no recurrence on the ESD scar (Fig. 4a). However, 74 months after ESD, surveillance colonoscopy showed an elevated lesion resembling a submucosal tumor with a central depression on the ESD scar (Fig. 4b). A biopsy specimen from a central depression showed group V. We therefore diagnosed the lesion as a local recurrence after ESD and performed a low anterior resection (intersphincteric) with lymph node dissection. Pathology revealed a well-differentiated adenocarcinoma, which was the same as the ESD specimen. The cancer was exposed to the mucosa at the central depression and infiltrated into the muscular layer (Fig. 5). There were no lymph node metastases. A local recurrence was diagnosed 9 months after surgery and the patient underwent an abdominoperineal resection and adjuvant chemotherapy. He is alive with no cancer recurrence.

## Discussion

ESD enables en bloc resection regardless of the size of the malignant lesion. It allows for adequate and detailed pathological examination and functional preservation of organs if curative resection is feasible by ESD [1]. Colorectal ESD enables endoscopic resection of tumors with a diameter >2 cm, a maximum limit easily resectable en bloc by endoscopic mucosal resection. In the future, one can expect an increase in the utilization of cancer resection by colorectal ESD.

A drawback of ESD, however, is that it involves a dissection and manipulation that is performed with the tumor exposed for a long period of time. This might lead to a risk of local recurrence due to implantation of tumor cells. In the present case, although pathology revealed a well-differentiated adenocarcinoma that was the same as the ESD specimen, we were unable to deny a new lesion histopathologically. Before ESD, colonoscopy showed no synchronous lesion in the rectum; however, it is impossible that a new lesion grew in such a short time period, because there was no evidence of a tumor in the colonoscopy performed 21 months earlier. Furthermore, Tanaka et al. [3] reported that proliferative activity of a recurrent tumor was significantly higher than that of a primary tumor. Nakajima et al. [4] reported that colonic carcinoma resembling a submucosal tumor is very rare and Masuda et al. [5] reported that local recurrence after endoscopic resection often shows a lesion resembling a submucosal tumor. Thus, we strongly suspected a local recurrence after ESD as opposed to a new lesion. Additionally, the tumor had been resected en bloc by ESD and the vertical and lateral margin was negative histopathologically. As the tumor was intramucosal, there should be no skip vascular invasion into submucosal layer. Therefore, we suspected that the local recurrence was due to implantation of tumor cells during the initial ESD.

Intraluminal lavage is an essential procedure during rectal cancer surgery to prevent recurrence at the anastomotic site due to implantation. A recurrence is thought to occur due to cancer cell implantation at the anastomotic site. These cancer cells exfoliate into the intestinal lumen during surgical manipulation. It has been reported that intraluminal lavage before anastomosis can help achieve a negative status for exfoliated cancer cells in the intestine and prevent recurrence at the anastomotic site [6–8]. When the rectum is mobilized in the pelvic cavity during rectal cancer surgery, cancer cells are exfoliated into the intestinal lumen during surgical manipulation. There are differences between cancers, often advanced cancers, treated by rectal cancer surgery and early cancers treated by ESD. However, if cancer cells exfoliate because of manipulation through the intestinal wall in rectal surgery, then tumor cells likely exfoliate into the intestinal lumen during ESD. In ESD, a knife is used for dissection as the hood compresses on the tumor while the tumor is exposed for a long period of time.

Reports in the literature suggest that for post-ESD local recurrences to occur by implantation, tumor cells have to exfoliate into the intestinal lumen and implant onto and grow on the dissection surface created during the ESD. For example, Umpleby et al. [9] reported the high viability of colorectal carcinoma cells that exfoliated into the intestinal lumen due to intraoperative manipulation. Morgan [10] reported that carcinoma cells that exfoliated into the intestinal lumen did not survive on the normal mucosa but did survive on a raw surface, such as that created by ESD dissection.

As in rectal lavage soon after rectal cancer surgery, intraluminal lavage after ESD is considered the most facile and effective method to prevent recurrence thought to be from implantation of tumor cells after ESD.

We report a case of local recurrence thought to be from implantation of tumor cells after ESD. Future studies are necessary to examine such cases of local recurrence after ESD, in order to better characterize the risk of implantation of tumor cells in this setting.
